# Conference report on the 14th International Society of Paediatric Oncology African Continental Meeting, 16–18 March 2022, Kampala, Uganda

**DOI:** 10.3332/ecancer.2022.1423

**Published:** 2022-07-07

**Authors:** Jaques van Heerden, Lisa Christine Irumba, Karim Assani, Julia Downing, Alan Davidson, Laila Hessissen, Judy Schoeman, Trijn Israels, Sharon Cox, Hafeez Abdelhafeez, Shauna Odongo Arao, Jeannette Parkes, Joyce Balagadde-Kambugu, Jennifer Geel

**Affiliations:** 1 Paediatric Haematology and Oncology, Department of Paediatrics, Antwerp University Hospital, Antwerp 2650, Belgium; 2 Paediatric Haematology and Oncology, Department of Paediatrics and Child Health, Faculty of Medicine and Health Sciences, Stellenbosch University, Tygerberg Hospital, Cape Town 7530, South Africa; 3 Department of Paediatric Oncology, Uganda Cancer Institute, Kampala, Uganda; 4 Palliative Care Association of Uganda, Kampala, Uganda; 5 International Children’s Palliative Care Network, Bristol BS1 2NT, United Kingdom; 6 Palliative care Education and Research Consortium, Kampala, Uganda; 7 Paediatric Haematology-Oncology Service, Red Cross War Memorial Children’s Hospital, University of Cape Town, Cape Town 7701, South Africa; 8 Paediatric Oncology Department, Children’s Hospital, University Mohamed V Rabat, 6533, Morocco; 9 Paediatric Haematology and Oncology, Department of Paediatrics University of Pretoria, Steve Biko Academic Hospital, Pretoria 0001, South Africa; 10 Queen Elizabeth Central Hospital, Kamuzu University of Health Sciences, Blantyre, Malawi; 11 Division of Paediatric Surgery, Red Cross War Memorial Children’s Hospital, University of Cape Town, Cape Town 7701, South Africa; 12 Department of Surgery, St Jude Children Research Hospital, Memphis, TN 38105, USA; 13 Department of Paediatric Oncology, Uganda Cancer Institute, Kampala, Uganda; 14 Department of Radiation Oncology, Groote Schuur Hospital and the University of Cape Town, 7701, South Africa; 15 University of the Witwatersrand, Division of Paediatric Haematology-Oncology, Charlotte Maxeke Johannesburg Academic Hospital, 2000, South Africa; On behalf of SIOP Africa and the Organising Committees of SIOP Africa 2022

**Keywords:** SIOP, Africa, Uganda, children, conference, workshops, WHO, six index cancers, innovation

## Abstract

Together with the Africa Continental Branch of the International Society of Paediatric Oncology (SIOP Africa), the Uganda Cancer Institute, a tertiary governmental institution for specialised cancer care services, research and training, hosted the 14th continental meeting of SIOP Africa from the 16–18 March 2022. Under the conference theme, ‘Innovate for Africa’, the hybrid conference brought together close to 400 international delegates to discuss innovations and experiences, as well as share the latest research in the field of paediatric oncology. The World Health Organisation 2030 Global Initiative for Childhood Cancer provided the main starting point for the conference with a comprehensive pre-conference workshop programme, from multiple stakeholders and organisations and the themes for the plenaries towards improving survival to the main breakout sessions. Delegates discussed various ways of improving outcomes in Africa, despite the challenges faced individually and collectively ranging from education, management systems and treatment guidelines to future governmental and NGO involvement in African cancer care. The main achievements of the conference were various commitments for collaboration, investing in junior investigators, development of registries and systems for improved childhood care on the African continent, while working towards greater access to advanced management options such as targeted therapies and bone marrow transplant services.

## Introduction

In March 2022, International Society of Paediatric Oncology (SIOP) Africa held the continental SIOP conference in Kampala, Uganda. The highly successful event, delayed for a year due to the COVID-19 pandemic, brought together colleagues and friends from 33 countries and 3 continents, confirming the desire for the paediatric oncology community to engage in-person. The conference was organised by the Uganda Cancer Institute (UCI), supported by the Ugandan Cancer Society, the Palliative Care Association of Uganda, the Uganda Child Cancer Foundation and the Uganda Paediatric Association. Colleagues from South Africa, Egypt and the USA supported the scientific committee and the organisers of the pre-conference workshops.

Since the 2018 launch of the World Health Organisation (WHO) Global Initiative on Childhood Cancer (GICC) aimed at reaching at least a 60% survival rate for children with cancer by 2030 [1], efforts on the African continent have been coordinated to reach these goals. The theme for the conference ‘Innovating for Africa’ was closely aligned with the WHO 2030 GICC agenda [2], and the scientific programme focused on practising paediatric oncology in African circumstances, advanced care on the continent, multidisciplinary and multinational collaboration.

In keeping with the theme of innovation, this SIOP Africa was the first hybrid meeting to facilitate access to delegates to participate regardless of their location, allowing international experts to share their expertise. A record number of pre- and post-conference workshops were held by colleagues from fields including medical, surgical, radiation oncology, allied health services and civil society. In line with SIOP International and SIOP Africa strategies, many of the educational programmes were aimed at improving content for junior investigators [3]. Echoing the WHO GICC guidelines, the conference was supported by the national government, backed by the Uganda Ministry of Health, and included plenaries with a focus on organisational and governmental structures to advance management in Africa.

### Conference statistics

The scientific committee received 140 abstracts from more than 35 countries. The in-person component attracted close to 400 participants from 33 countries, with the majority coming from Uganda, South Africa, Ghana, Ethiopia and Tanzania. Disciplines represented were paediatric oncology, paediatric surgery and neurosurgery, nursing, dietetics, pharmacy, rehabilitation, pathology, social work and psychology. Scholarships were awarded to 123 medical and nursing delegates. The virtual component, generously sponsored by St Jude Global and run by Cvent, attracted 55 viewers.

## Main scientific conference

### Opening address

SIOP Africa President Joyce Balagadde-Kambugu referred to the WHO GICC goals in the context of multiple challenges on the African continent, requiring locally relevant and innovative solutions and hence the theme ‘Innovate for Africa’. SIOP Africa is dedicated to developing systems and opportunities in Africa to achieve these goals, including engagement with governments. These innovations should improve access to diagnosis, treatment, supportive and palliative care for children with cancer and ensure the gains made are not lost.

SIOP President Kathy Pritchard-Jones commended the paediatric oncology community on their response to the COVID-19 challenges, continuing to apply best practice standards of care with necessary adaptations and collaborating on clinical research studies that improve access to treatment. The topics of the pre-conference workshop, in particular, the adapted treatment regimens, provided an opportunity to discuss the realities of implementing the WHO’s GICC. Good infrastructure and multidisciplinary care are prerequisites for tackling the unmet needs for cancer control, including childhood cancer control. The SIOP President-elect Guillermo Chantada praised SIOP Africa in leading the way in adapting therapies to local resources, addressing specific regional needs and adapting innovations for the advancement of paediatric oncology.

The WHO representative to Uganda Yonas Tegegn Woldemariam commented on the difficulty in contextualising African paediatric oncology cases in the 400,000 annual global childhood cancers, because the burden of childhood cancers in Africa is underestimated due to poor coverage of population-based cancer registries. The inadequate capacity for early detection of cancers, including childhood cancers, compounded with limited treatment capacity, are the main drivers of low cancer survival rates in Africa. Reliable and accurate epidemiological cancer data to inform cancer control services delivery with adequate human resource capacity were central to improving national cancer control.

The keynote address, ‘The history of cancer in Uganda’, was presented by Charles Olweny, UCI Board Chair, detailing how paediatric cancer services began as lymphoma treatment centres at the Mulago National Referral Hospital where Dennis Burkitt first described Burkitt lymphoma. This centre grew to become the present UCI. The concept of essential oncology drugs in Africa, started in Uganda, has demonstrated that we can improve access to essential cancer drugs in Africa. The concept originated from the essential drugs list, where utilising a limited number of medications, most diseases could be treated effectively. The same principle was applied to chemotherapy drugs such as vincristine, doxorubicin, cyclophosphamide etc. [4]. By ensuring the availability of these essential chemotherapy drugs for the six index childhood cancers in all African countries, the GICC goals become more reachable.

Jackson Orem, Executive Director of UCI, reflected on the successes of the Uganda Cancer Institute which was mandated by an act of parliament to establish four regional cancer centres and has completed the development of a national cancer control plan. His reflections brought home the importance of government support in the establishment of training programmes for cancer specialists, including paediatric oncologists. UCI, which receives 7,000 new cancer cases, 10% of whom are children, continues to strive to improve clinical and scientific services with the Integrated Electronic Medical Record System, a fully automated radiology unit and laboratory auto-analysers. Jane Ruth Aceng, Minister of Health for Uganda, emphasised the importance of the government in fighting childhood cancer on a continent with low survival rates of less than 30% in many countries. She attributed Uganda’s survival rate of approximately 50% to leveraging resources for cancer control with truly mutually supportive partnership focusing on similar goals.

### Plenaries

#### WHO Global Initiative for Childhood Cancer (GICC)

Childhood cancer mortality reflects global disparities in health outcomes. Avoidable deaths from childhood cancers in low- and middle-income countries (LMIC) result from, among others, failure to prioritise early detection, misdiagnoses, lack of access to care, treatment abandonment and treatment delays. Inequities in quality care outcomes for paediatric cancer in LMICs demand urgent engagement of national and regional government structures to accelerate improvement in paediatric cancer continuum of care. The WHO GICC lays out policy advocacy, including childhood cancer policy and plans, for improved childhood cancer care outcomes. The WHO’s proposed technical ‘CureAll package’, includes the creation of centres of excellence; universal health coverage; regimens and roadmaps for diagnosis, treatment, evaluation and monitoring; and a sufficient competent workforce. The three cross-cutting enablers are advocacy, leveraged financing and linked policies/governance. SIOP President Kathy Pritchard-Jones noted that even with the establishment of multiple Paediatric oncology units (POUs) in large countries, all can deliver quality care. She recommended the term ‘Centres of Expertise’ rather than ‘Centres of Excellence’ to avoid discouraging those who have not been classified as ‘Centres of Excellence’, to encourage development of specialist centres, but not to distinguish on the basis of the quality of care rather the level of care.

#### ‘Not without us’—Advocacy for access by patients, survivors and parents—Focus on Uganda

Limited financing for cancer control and early detection remains a bottleneck in LMICs like Uganda. Uganda developed their first Uganda National Cancer Plan (UNCCP), through a wide consultative process coupled with active participation and involvement of various stakeholders. In this UNCCP, a section is dedicated to childhood cancer control, with the main goal to achieve an overall survival of 60% for children with cancer by 2026. Advocacy for cancer control is required in all resource settings, but should be a consolidated effort to avoid fragmented approaches. Stigma towards people with cancer should be addressed as a human rights issue. This may be achieved by involving people with cancer and survivors in cancer control advocacy towards improving advocacy and service delivery. An example of efforts to increase awareness and decrease stigma is the 3C club (Children Caring about Cancer), a peer-to-peer network.

#### Targeted therapy

Alaa Elhaddad (Egypt) focused on the international application of precision medicine. Opening those increased efforts was needed to develop this modality on the African continent. The challenges of targeted therapy in clinical practice in Africa include high cost, long turnaround time, failure to obtain adequate tumour material, defining pathogenic variants in paediatrics and ethical issues surrounding genetic material. Paediatric malignancies have a relative paucity of targeted mutations and distinct molecular alterations compared to adult cancers, and rational combinations of targeted therapies are required in the universalisation of practice.

Jennifer Geel (South Africa) described increasing crowdfunding efforts to afford targeted therapies for individuals in South Africa, suggesting that potential funders be informed of the expected poor prognosis of many of these patients. Pharma-sponsored clinical trials for targeted cancer therapy for children in South Africa may increase access, but care should be taken to protect vulnerable populations and patients.

Suzanne Turner (UK) highlighted Africa’s role in the development of targeted therapy for conditions such as lymphomas. The potential unique genetics and biology of lymphoma in Africa are under study in collaborative projects, and efforts are underway to identify existing efficacious drugs with fewer side effects with patient surrogate mouse models. Turner stressed that targeted therapies must be adapted to the environment in which they are administered.

Mahmoud Hammad (Egypt) presented chimeric antigen receptor (CAR) T-cells therapy as a viable treatment option in Egypt for children and young adults to achieve remission in relapsed/refractory B-ALL. Barriers to CAR T-cell therapy include resource constraints such as cost, expertise and infrastructure. Modified protocol development and twinning are required to support Africa in the development and implementation of CAR T-cell therapy. Support should be given to this therapeutic modality while financing and developing infrastructure.

The panel discussion concluded with the recommendation that more bone marrow transplant specialists should be trained, with the Children’s Cancer Hospital (Egypt) being a viable option as a centre with high volumes [5].

#### Childhood cancer in special circumstances (refugees, internally displaced persons and other humanitarian crises)

Ugandan WHO Country Representative Yonas Tegegn Woldemariam focused on poor healthcare systems which influence childhood cancer survival. Disasters disrupt healthcare for both patients and healthcare workers. There is a need to advance universal health coverage, address health emergencies and increase government investment, continental cooperation and global partnership for technology transfer and capacity building. The African continent should accelerate the adoption and implementation of childhood cancer initiatives and related tools.

Francine Kouya (Cameroon) shared her experience in a conflict zone, stressing the importance of patient follow-up programmes, strengthening multidisciplinary teams and the value of communication for patient safety. In times of crisis, psychosocial support has increased importance to parents, children and healthcare workers involved in childhood cancer care.

Access to basic palliative care is still lacking for refugees according to Eddie Mwebesa (Uganda) and a palliative care policy should be implemented to improve the management of refugees with cancer. Provision of food, shelter and education often takes priority over healthcare in refugee settlements, with a negative impact on the survival of children with cancer.

Julius Ecuru explored ethical considerations in the management of these vulnerable children, whose treatment may be interrupted or delayed due to visa and working permit applications and lack of resources to pay for medical services. Potential solutions would be for host countries to anticipate special circumstances, prepare for their management, embrace humanitarian holistic care for these children and use technology to help in hard-to-reach areas for the provision of health services.

## Conference presentations

### Medical tracks

Margaret Lubwama (Uganda) reported high rates of multidrug-resistant bacteria, the main cause of bacteraemia in paediatric haematologic cancer patients with febrile neutropaenia, while Youssef Madney reported that Echinocandin-resistant *Candida* infections were a source of high mortality in haematological malignancies in Egypt [6]. El-Mahallawy reported that rapid molecular detection of organisms with real-time multiplex PCR detection during sepsis could improve outcomes by guiding more targeted antimicrobial use. Use of this modality, which detects 21 different bacterial pathogens, resulted in a significantly shortened time to detection of bacterial pathogens with rapid change to appropriate antimicrobials.

Christin Edan (France) described the successful initiation of a 3-year palliative care programme integrated into POUs in 15 Francophone countries under the auspices of the French-African Paediatric Oncology Group (GFAOP).

Both Lily Gloria Tagoe (Ghana) and Mapule Kholong (South Africa) reported low rates of COVID-19 in children with cancer, although Tagoe reported 2 deaths in a cohort of 10 infected patients, while Kholong reported minimal clinical impact on 432 patients tested. Rose Nankinga (Uganda) described the increased rate of late presentations due to the pandemic, threatening to reverse gains made in overall survival, and Karim Assani from the AMCC group indicated that the pandemic increased the difficulties of implementing programmes aimed at improving the survival of patients with retinoblastoma.

In Tanzania, pupillary dilatation, high intraocular pressure, shallow anterior chamber and extensively necrotic tumours were associated with histopathological high-risk features in retinoblastoma, according to Neema Moshi, and Nicholaus Benedicto found that Tanzanian parents or caregivers refused enucleation because of their perception towards the appearance of the child after enucleation, low level of education, traditional and religious beliefs and poor socio-economic status.

Ernestina Schandorf reported a 5-year OS for nephroblastoma in Ghana for stages 1 through to 4 were 100%, 87.7%, 71.1% and 52.4%, respectively. Sahmima Namugerwa (Uganda) linked higher survival in nephroblastoma to improved pathology reporting, increased intensity treatment and improved access to surgery and radiotherapy.

On behalf of the South African Neuroblastoma Working Group, Jaques van Heerden reported that less than half of the patients with high-risk neuroblastoma (HR-NB) are operated on, mostly determined by post-induction metastatic remission rate, with non-standard surgical practices, leading to variable OS. A greater rate of primary tumour resection in HR-NB is advocated to improve survival. Robyn Charlton (South Africa) found that socio-economic factors were not significantly associated with neuroblastoma survival, suggesting that tumour biology exerts an overriding influence on prognosis. Irene Nanyanga (Uganda) demonstrated that palliative metronomic CADo led to longer median survival time.

Brenda Mallon for the GFAOP group showed the feasibility of staging and estimating outcomes for Burkitt lymphoma, retinoblastoma and nephroblastoma, according to the Toronto Paediatric Cancer Staging guidelines, in seven hospital-based cancer registries.

Richard Nyeko (Uganda) demonstrated the importance of multidisciplinary team meetings in the improvement of brain tumour care in Uganda, while Mwebe Katasi described the capacity to improve the early detection, referral, diagnosis and comprehensive care for children with brain tumours in low-resource settings through innovative collaborations.

Chemotherapy utilisation (reliable supply chain, safe chemotherapy preparation, administration and disposal) is suboptimal in LMICs such as Ethiopia. Atalay Mulu Fentie identified gaps and designed interventional strategies at POUs to improve safety. In Ghana, most antineoplastic medicines surveyed were found in the private pharmacies; however, the mean availability across all studied pharmacies was below the WHO target of 80%. Kofi Boamah Mensah concluded that the low availability of medicines at public pharmacies indicates the need for government interventions. Furthermore, Ghanaian community pharmacists play an essential role in the provision of cancer health promotion services. Shauna Aroa (Uganda) audited the UCI chemotherapy safety programme, leading to the establishment of a multidisciplinary team with pharmacist involvement. This allowed formalisation of chemotherapy prescribing and administration, which highlighted the value of paediatric pharmacy input for safety, standardisation and education of staff and patients.

## SIOP global mapping programme

A comprehensive overview of paediatric oncology care in Africa from the SIOP Global Mapping Programme emphasised marked disparities between countries. Some countries have highly specialised services, while no paediatric oncology services are present in certain countries, including Mauritius, until recently a high-income country [7]. A long-term strategy to eliminate disparities in African paediatric cancer care should be aligned with the WHO GICC aims and facilitated by SIOP Africa. Patrick Makupe (Malawi) reported that only 36 of 47 reporting countries had physiotherapy services available to children, with higher availability in upper-middle-income countries. As survival rates improve in LMICs, it is vital to increase the awareness of paediatric oncology rehabilitation needs and attract/retain rehabilitation professionals. Mawethu Bell (South Africa) stated that the majority of reporting African POUs have access to social workers, while a minority access other professionals involved in psychosocial care, placing a disproportionate burden of responsibility on social workers. Investment in all components of psychosocial care is strongly recommended to contribute to improvements in childhood cancer care and survival in Africa. The SIOP Global Mapping Programme data also confirmed that the number of population-based registries remains stable at only 6 in Africa, while only 17 countries reported hospital-based registries. Responses from nine countries indicated that they had a national cancer registry, a paediatric oncology registry and a national paediatric oncology association, suggesting that the minority have the organisational infrastructure to coordinate strategic efforts in childhood cancer care. Nursing data confirmed gross understaffing and priority research and training topics were identified, including professional practice, psychosocial support, chemotherapy administration and side effects, psychosocial support, palliative care, infection prevention and control. The theme that emerged from the Global Mapping Programme presentations was that coordinated efforts are both required and possible to introduce low-cost and high-impact interventions to achieve WHO GICC goals.

### Nursing track

The nursing track comprised 2 full days of free paper sessions attended by over 80 nurses from more than 30 countries. Nursing training was highlighted by Elianeth Kiteni (Tanzania) due to the heterogeneous training nursing staff receive in paediatric oncology. The SIOP Nursing baseline standards have been introduced in an effort to formalise paediatric oncology nursing training. Joan Nakabiri (Uganda) and Tadala Mulemba (Malawi) presented the Global HOPE nursing education programme as an example of continued professional education for nurses. Wendy Eyiah-Mensah described the initiation of a specialist paediatric oncology nursing programme in Ghana, a collaboration between partners in the UK, USA and Ghana, resulting in 17 graduates in the first year.

Glenn Afungchwi (Cameroon) described 57 topic areas identified for inclusion in a foundation course, including a general introduction to cancer and treatment modalities; chemotherapy administration and side effects; psychosocial support, palliative care and infection prevention; and control. Nurses were involved in the development of five (23.8%) national cancer control plans with specific recommendations for nurses. During the COVID-19 pandemic, nurses were forced to adapt to online training. The ‘Fundamentals in Paediatric Oncology’ programme, developed by the GFAOP nursing group in 2013, evolved into an e-learning format with 10 modules. Continuous weekly nursing education sessions at UCI have improved nurses’ knowledge and attitudes, are sustainable, cost-effective and further multidisciplinary team integration. The Global HOPE programme continues to develop nurse leaders from Botswana, Malawi and Uganda in a year-long programme. Modules include essential nursing leadership skills, high-performance teams, diplomatic communication, strategic management and practice models, and nursing quality care. Paediatric palliative care training of healthcare providers in resource-poor settings has been prioritised, with 52 graduates of an online course run in collaboration with Global HOPE.

Invited speaker Marilyn Hockenberry emphasised the use of protective gear as well as the important role played by nurses in cross-checking and confirming prescriptions prior to chemotherapy administration to minimise treatment errors. Enyo Bosumprah described a teaching tool to empower nurses to educate parents on discharge on important issues such as response to fever, take-home medication and treatment side effects. The tool resulted in marked increases in test scores, which may translate to improved patient outcomes.

### Civil society track

This track successfully incorporated the presentations from various members of the multidisciplinary team, fostering goodwill and networking. Glenn Afungchwi reported that effective training of healthcare workers on early warning signs of childhood cancer required understanding cultural beliefs and values. Both Nchasi *et al* and Gategetse *et al* reported that in-person on-site training was effective for increased awareness on early warning signs of childhood cancer. The Awareness for Burkitt Lymphoma Eradication programme (ABLE+) in Northern Uganda is an example of a successful intervention to increase detection and referral, resulting in a marked increase in referrals of children with Burkitt lymphoma.

Treatment abandonment remains an obstacle to cure in sub-Saharan Africa, where cost is an overriding reason for abandonment. George Chagaluka (Malawi) described a comprehensive package to enable parents to complete treatment. Results from a World Child Cancer (WCC) survey indicated that in Ghana, the majority of respondents felt that they would not have been able to access treatment for their child without NGO support, and the most important type of support included financial support for drugs and diagnostic tests.

Claire Namulwa (Uganda) reported that sustained psycho-social support retained children in cancer care during the COVID-19 lockdown and ensured completion of treatment, highlighting the importance of psychosocial care in adherence and raising survival rates. A study of rural Ugandan adolescents reported fear of death which felt inevitable, fear of dependency and lack of privacy, and fear of being a burden. They expressed the desire to be given the opportunity to make decisions relating to their own care, despite parental opinions that they are incapable. Depression and isolation were common feelings, exacerbated by isolation from peers and mutilating surgeries such as amputations. Adolescents reported that peers questioned their ability to function as sexual beings, and they reported stress due to fear of infertility. A Kenyan study on gaming technology as a method of non-pharmacological pain relief noted that in 75% of the cases, cooperation during procedures was improved. Other outcomes included more engagement with peers and improved coping skills. Chagira *et al* suggested that children’s hospitals should incorporate a variety of gaming technology as tools within greater play-based oncology programmes to improve overall coping and psychosocial care for children with cancer. From a study of nurses’ well-being, four interventions were highlighted as areas where nurses need greater support: access to a psychologist, case study meetings to discuss emotional impact of work, greater support and recognition from management and more training in emotional well-being and resilience.

### Conference awards

Lifetime and academic awards awarded after the conference can be seen in Table 1.

## The pre- and post-conference workshops and symposia

An overview of the workshops and symposia can be seen in Table 2.

### 1. Alliance Mondiale Contre le Cancer (AMCC) Retinoblastoma Workshop Day

The ‘Alliance Mondiale Contre le Cancer’ (AMCC) is an NGO mainly focused on women’s and children’s cancer in LMICs through training, education and research [8]. The AMCC retinoblastoma workshop was designed to support multidisciplinary teams managing retinoblastoma to achieve early diagnosis, access to treatments, rehabilitation and follow-up of children in sub-Saharan Africa. The aim of strengthening networks between teams treating retinoblastoma in English-speaking and Portuguese-speaking sub-Saharan Africa was successful as 105 delegates shared experiences of achieving early diagnosis, discussing aspects of patient care, data collection and research. The group of professionals included 36 ophthalmologists, 11 ocularists, 26 paediatric oncologists, 10 pathologists and 20 ophthalmic officers/nurses from 14 countries.

Guillermo Chantada (Argentina) discussed different therapeutic approaches according to settings [9]. Didi Fabian (Israel) spoke about disparities in treatment outcomes, based on large global retinoblastoma data sets [10, 11]. Laurence Desjardins (France) provided baseline data on retinoblastoma management and outcomes in Anglophone Africa. Presenters from Uganda, Rwanda, Ghana, Nigeria, Tanzania, Kenya, Ethiopia and Mozambique shared local experiences, providing rich material for discussion in both plenary and breakaway sessions. Emphasis was placed on the importance of ocularists providing prostheses to enhance cosmesis and quality of life, thus limiting stigmatisation while improving adherence and survival [12].

The workshop compiled an early diagnosis multi-year plan with the agreement of the Ugandan Ministry of Health. Aims included that 1) all teams in attendance develop multidisciplinary care of children with retinoblastoma and organise at least one referral centre per country for conservative treatments to preserve eye and vision in bilateral cases; 2) all teams collect data on a common basis for all cases of retinoblastoma in their respective countries; and 3) all teams advocate for the reduction of the cost for retinoblastoma care because no child should die of a highly curable malignancy, such as retinoblastoma, because of financial barriers.

The workshop concluded with an invitation to all teams to participate in the regular webinar every 2 weeks to discuss challenging cases.

### 2. Civil Society and Parents Symposium

The Civil Society and Parents Symposium attracted 57 professionals and individuals across the continuum of childhood cancer care to discuss initiatives to benefit children’s treatment experiences. Emphasis was placed on patients as the primary reason for discussion of improvements in childhood cancer care, rehabilitation and reintegration of survivors. The theme ‘Nothing for us without us’ sent a strong message that patients and caregivers should be integral to the decision-making and planning of childhood cancer services. Some of the key talking points included getting feedback from families that are taking care of cancer patients, psychosocial issues experienced by adolescents, treatment abandonment, retention in care, psychosocial support for vulnerable children with cancer during COVID-19 lockdown, need for educating healthcare workers on the early warning signs of childhood cancer and national paediatric oncology registries in Africa.

The symposium acknowledged the position that childhood cancer care may not succeed without parents and caregivers supporting and providing for the children during the process of receiving care. Attendees from diverse backgrounds were encouraged to address specific gaps according to their skill sets, expertise and passion. As many civil society organisations were formed as a result of individuals and groups who have lived through the experiences of childhood cancer itself, their particular experiences were acknowledged as vital to quality care for children and adolescents with cancer.

Recommendations included the need for increased advocacy for budget increment by governments, increased efforts directed at developing or creating an enabling policy environment for childhood cancer care and management and public-private partnerships to accelerate access to care. Suggestions included increased patient involvement, especially cancer survivors, in advocacy efforts and involvement of schools.

### 3. IPSO Surgical Symposium

The International Society of Paediatric Surgical Oncology (IPSO) [13] hosted its first meeting as part of the SIOP Africa conference, its first truly hybrid meeting. Presentations focused on surgical principles and challenges in various paediatric solid tumours treated in African centres, sparking robust discussion and engagement [14]. An inspiring talk entitled ‘Paediatric surgical oncology in practice in Uganda: A journey of 20 years’, by Arlene Muzira and John Sekabira, highlighted both the growth of the paediatric oncology service in the country and the current limitations of local resources. Sharon Cox discussed vascular access including appropriate line choice and modifications in techniques applicable to LMIC settings.

Nasser Kakembo (Uganda) highlighted the adaptation of resources to achieve optimal outcomes in surgical management of nephroblastoma. Derek Harrison’s presentation on bilateral nephroblastoma recommended neoadjuvant chemotherapy for at least 6 weeks, or 12 weeks in cases of suboptimal response, followed by bilateral nephron-sparing surgery, if possible, or unilateral nephrectomy with nephron-sparing surgery in the least affected kidney. In cases of nephroblastoma with inferior vena cava extension, the recommendation is to administer neoadjuvant chemotherapy for a maximum of 6 weeks, following which infrahepatic thrombus may be removed safely, while suprahepatic disease requires cardiopulmonary bypass on standby while thrombus is removed from the right atrium.

Hafeez Abdelhafeez (USA), a surgeon with experience in both low- and high-income settings, stated that the optimal setup for safe hepatoblastoma surgery is feasible across different settings. Peri-operative optimisation, inflow and outflow control, a parenchymal transection strategy, haemostasis and biliostasis are key steps in mitigation of complications.

Hafeez discussed the principles of sarcoma staging, biopsy and management and sound tumour biopsy strategy. Understanding the role of multimodality therapy and neoadjuvant therapy based on tumour biology and anatomy and complete surgical resection with preservation of form and function are key factors to improving sarcoma outcomes.

Jed Nuchternon spoke on the principles of neuroblastoma staging and management, emphasising multimodality therapy, indications for resection, extent of resection and operative considerations.

A ‘tumour board’ interactive panel discussion took place where the management of five patients was debated. This interactive session proved to be the highlight of the meeting with the principles from the previous talks being put into practice, creating lively debate. The day was very well attended with delegates from many continents and many African countries and allowed old and new friends a chance to catch up and share their experiences, be it anecdotal but valuable experiences or sharing new cutting-edge practices based on latest protocols and guidelines. Due to the unprecedented success of this inaugural meeting, IPSO plans this to be the first of many African continental meetings going forward, and we all look forward to meeting up again in the near future.

### 4. Nurses Children’s Palliative Care Workshop

A one-day workshop on children’s palliative care was coordinated by the International Children’s Palliative Care Network (ICPCN) [15], with experienced facilitators from ICPCN, the Palliative Care Education and Research Consortium [16] and WCC [17]. The interactive day provided opportunities for discussion and sharing of experiences. The workshop introduced participants to the principles and practice of palliative care and its integration into the management of children with cancer. Topics included pain assessment and management, communication, managing other symptoms, advanced care planning, end of life care, the nurse’s role and caring for ourselves. Eighty nurses attended the conference from a range of countries, including Uganda, Rwanda, Zambia, Tanzania, Kenya, South Africa, Ghana, Malawi, Egypt, Sudan, Nigeria, Cameroon, Zimbabwe, UK and Sweden. Key messages included 1) that palliative care is an integral part of the care of children with cancer; 2) that we can manage pain and symptoms effectively; 3) that communication is key, as is how we discuss diagnosis and prognosis with children and their parents; 4) nurses have a key role in the provision of palliative care for children with cancer; and 5) that in order to provide palliative care for children with cancer, we need to care for ourselves and be committed to developing our resilience. The workshop was made possible through funding from the Burdett Trust for Nursing.

### 5. Nutrition Workshop

Approximately 46% of the children with cancer in Africa are malnourished at diagnosis [18]. Malnutrition in children with cancer is associated with increased infection, toxicity, decreased survival and increased abandonment of care [19]. Through collaboration with the International Initiative for Paediatrics and Nutrition (IIPAN), significant advances have been made in improving the education and clinical capacity of nutrition services in Africa, closing the gap on several unmet needs reported by paediatric oncology units in Africa [20]. To further expand on this regional initiative, a post-conference nutrition workshop was held with the aim to improve knowledge on the delivery of care and management of nutritional complications among children with cancer. This workshop reviewed fundamentals of nutrition assessment and intervention, providing instruction on the management of nutritional conditions common in childhood cancer, which may be particularly challenging in LMICs. Attended by approximately 50 participants (live and virtual) the audience included dieticians, nutritionists, nurses, physicians, parent groups and non-governmental organisations.

Elena Ladas (USA) provided an overview of IIPAN activities in Africa and Judy Schoeman (South Africa) described the objectives of the SIOP Global Health Nutrition Workgroup such as advocating for nutrition as an essential member of the management team, increasing nutritional education on the African continent and increasing dieticians and nutritionists in LMICs. IIPAN nutritionists from Cameroon, Tanzania, Uganda and Kenya presented cases relevant to local settings and optimal delivery of nutritional care with limited resources, while paediatric oncologists presented the management of severe acute malnutrition, mechanisms of liver impairment and nutritional management of enterocolitis/pancreatitis. Happiness Ndifon (Cameroon) discussed the role IIPAN played in establishing food supply during COVID-19 lockdown in 2019–2020. In summary, the workshop highlighted important nutrition-related management challenges that need increased attention in the African setting in parallel to building the capacity of nutritionists and dieticians on the continent.

### 6. Paediatric Oncology Pharmacy Workshop

True multidisciplinary care for children with cancer includes a pharmacist for safe preparation, administration and rational use of anticancer and supportive care medications. In LMICs, frequent stock-outs require precise pharmacy prognostication, ordering and tracking of medications. Pharmacists are critical for safe handling and disposal of medications and educating the medical team about new medications. However, the highly specialised field of Paediatric Oncology Pharmacy is poorly represented in most LMICs [21].

The aim of the inaugural SIOP Africa pharmacy workshop was to increase the involvement of pharmacists in the African paediatric oncology community. The workshop was attended by 31 people including survivors, herbalists, clinical pharmacists, paediatric oncology pharmacists, a clinical pharmacist with bone marrow transplant experience and paediatric oncology nurses. The pharmacy workshop explored the issues of mentoring, continuous professional development, specialisation, involvement in a multidisciplinary team and imparted skills in pharmaceutical care plans, safe chemotherapy administration and research [22].

Talks were given on paediatric pharmacy, off-licence and off-label use of drugs, clinical trials, nutrition, herbal medicines and cancer registries [23]. Friendships and collaborations with pharmacists, nurses and oncologists from Egypt, Ethiopia, Tanzania, Zimbabwe, Cameroon and South Africa were part of the successes of the workshop. Discussion of potential projects resulted in analysing the inclusion of pharmacists in paediatric oncology units across Africa, developing neutropaenic guidelines and regular education sessions to discuss a particular disease area, medicines involved and the role of the pharmacist.

Pharmacists have a vital role to play in the day-to-day management of cancer in children. The workshop was a call that united pharmacists from all over the continent, with the same goal to provide quality care to children with cancer. It is hoped that the momentum from this meeting will grow to have an important impact on childhood cancer survival rates in Africa.

### 7. Radiation Oncology Workshop

A one-day, online radiotherapy workshop was arranged by the SIOP Africa radiotherapy EXCO representatives, endorsed and supported by the International Paediatric Radiation Oncology Society (PROS) [24]. Registration for the workshop was free, allowing more participants to attend.

Twelve practical talks were aimed at radiation oncologists in Africa who are currently treating children. These included general considerations for treating children, modifications required to treat children in a busy adult radiotherapy centre, how to understand and incorporate paediatric disease-specific protocols, the role/interplay of the paediatric oncologist when treating children with radiotherapy in LMIC, imaging, anaesthesia for radiotherapy, best practice contouring, late effects and disease-specific guidelines for nephroblastoma, medulloblastoma, rhabdomyosarcoma and palliative care. International faculty from PROS and CCLG, based in the United Kingdom and the United States, assisted African faculty with practical talks.

The workshop proceeded seamlessly with 46 attendees from not only Africa, but globally. Informal feedback suggested that current challenges were addressed. This is just one example of how COVID-19 has facilitated improved online communication, with additional exposure to a relatively rare sub-speciality.

### 8. The Adapted Management Guidelines Workshop

The resources to develop AMG in Africa are not fully developed [25]. The AMG workshop was organised by SIOP Africa and St Jude Global as an interactive activity led by African and international experts who have developed resource-based management guidelines and protocols in their own settings. Participants were guided in the development process, stimulating problem-solving to develop locally relevant guidelines and protocols. A practical approach was used to initiate the development of treatment approaches for the multidisciplinary team, independent of tumour type or resource setting. The experiences of the attendees were used as the starting point to demonstrate that African healthcare workers are resourceful and can solve childhood cancer problems on the African continent that collaborative partnerships can advance knowledge and create insight for more innovative solutions and that interregional collaboration is of great value.

The 62 participants and 14 facilitators were from 22 African countries, 5 European countries and the USA, representing 58 institutions. The disciplines represented (paediatric oncology, paediatric surgery, ophthalmology, pharmacy, policy and nursing) were an example of the multidisciplinary collaboration required to successfully formulate AMGs, and participants left the energetic meeting with renewed vigour to continue this important work on their return home. The participants were equipped with the basic tools to identify available resources, assemble a multidisciplinary team and initiate discussion for the development of tumour-based protocols and clinical research questions.

### 9. Wilms’ Tumour Group/CANCaRe AFRICA Workshop

The Collaborative African Network for Childhood Cancer Care (CANCaRe Africa) is an active multidisciplinary regional network in sub-Saharan Africa [26–28]. It functions as a platform to improve outcomes of children with cancer in Africa through research, capacity-building and clinical care. The mission is to improve survival for children with common and curable cancers in sub-Saharan Africa by reducing treatment abandonment and death during treatment to less than 10% and by developing, implementing and evaluating locally appropriate treatment guidelines. The 13 participating centres are from Cameroon, Ghana, Malawi, Uganda, Kenya, Zimbabwe, Tanzania and Ethiopia.

At the workshop, the specific goals for the current projects were articulated: the Wilms’ tumour Africa project, ‘SUCCOUR – Supportive Care for Children with Cancer in Africa’ and ‘Zero Abandonment from Start to Finish’. The workshop was attended by site leads and steering committee members of CANCaRe Africa and facilitated by advisory committee members. Goals were articulated in the areas of research and data collection, teaching and education, advocacy, partnerships and capacity building. The goals include were to have over 95% complete data, to have a multidisciplinary symposium on a relevant supportive care topic every year, to strengthen the global alliance for the prevention of treatment abandonment with a focus on out-of-pocket costs for families and to continue to build local capacity with a focus on creating leadership roles for junior faculty. The intent is to publish the goals on the CANCaRe Africa website once finalised and approved by all site leads.

**The Wilms Africa Phase II study** started in January 2020 and is evaluating a revised and comprehensive adapted treatment guideline. In Phase I, end of treatment survival increased from 52% to 69% (*p* = 0.002) and treatment abandonment decreased from 23% to 12% (P < 0.001) [29].

**The SUCCOUR Phase II study** includes a clinical research and nursing component which aims to improve supportive care and the management of fever and neutropenia by implementing and evaluating a local care pathway. SUCCOUR Phase I reported on treatment-related mortality and demonstrated a high mortality of fever and neutropenia episodes and late start of empiric antibiotics, often without obtaining a blood culture [30, 31].

**The ‘Zero abandonment from Start to Finish’ study** aims to reduce treatment abandonment to less than 10%. The study includes a clinical research, advocacy and fundraising component to reduce out of pocket costs for families of children with cancer. Previous work supported the significance of preventing treatment abandonment to improve survival and the importance of reducing costs for families to enable them to complete the treatment of their child [32].

### 10. World Child Cancer Workshop

The WCC [17] workshop highlighted efforts to improve childhood cancer management in sub-Saharan Africa. The discussions focused on the main challenges, successes, lessons learned and plans for the future.

Sumit Gupta (Canada) demonstrated the case for cost-effectiveness of childhood cancer interventions in Ghana. This model, developed by the Policy and Economics Research in Childhood Cancer [33] unit, shows the cost of treatment per patient compared to daily-adjusted life year saved and compares this to the gross national income (GNI) per person where the cost per DALY is less than the GNI per capita; the treatment is cost-effective, as was demonstrated in Ghana [34]. Spending a little more money might improve outcomes and further increase the cost-effectiveness of the interventions.

Lily Gloria Tagoe provided a Ghanaian perspective on childhood cancer management. She shared the history and major achievements since 2010, including (1) an increase in the number of trained medical, nursing and pharmacy specialists; (2) the development of national treatment guidelines and improvements in supportive care, leading to improved survival of children with some of the GICC-focus tumours; and (3) initiatives to decrease treatment abandonment, such as the establishment of funded family accommodation near the paediatric oncology units. Before the launch of the Ghanaian National Childhood Cancer Strategy, the Ministry of Health announced that drugs for four common childhood cancers would be purchased by the government.

WCC efforts to improve outcomes in childhood cancer and the patient experience through strengthening paediatric oncology nursing were presented by WCC board members Rachel Hollis, Glenn Mbah and Ayire Emmanuel Adongo. They detailed components of the nursing education programme from the foundation phase through to training the trainers. A critical achievement was to obtain a commitment by the Ghana College of Nurses and Midwives (GCNM) to establish an accredited specialist programme in paediatric oncology nursing, resulting in the graduation of 17 nurse specialists in paediatric oncology in 2021.

### 11. Young SIOP Educational day

Systematic development, implementation and evaluation of standardised care practices are essential prerequisites to building a foundation for multicentre clinical research and incorporating these research discoveries into routine care. The recognition that African research is poorly represented on the international stage led the hosts of the first Young SIOP Education Day at the SIOP Africa conference to target emerging researchers on the continent. The event was held in hybrid format, hosted by the University of the Witwatersrand and Texas Children’s Hospital Global HOPE Programme. The theme ‘Standardisation of care as a foundation for paediatric cancer cooperative group research in Africa’ reflected the position that clinical research is a critical driver of quality care, novel therapies and quality of care to cure childhood cancer. Subject experts provided comprehensive reviews of the six GICC index cancers, building on historical overviews of developments in each cancer, with critical insights into latest trends in both high and LMIC settings. An afternoon session covered major points in research methods, with a particularly African flavour, such as ‘Approaches and strategies to integrate clinical research into busy clinical workflows’, which stressed persistent hard work to achieve success in research, and ‘Writing abstracts for presentations and publications’, which emphasised the importance of following simple rules to increase the chances of abstract acceptance at international conferences.

### 12. Francophone-African Group of Paediatric Oncology (GFAOP) meeting

The GFAOP was founded in 2000 by African and French doctors as a medical association bringing together a network of childhood cancer specialists from 18 countries of the Maghreb and sub-Saharan Africa with a vision that children with cancer in Africa can and should be treated locally by trained staff.

The GFAOP workshop entitled ‘Decision-making in the treatment of childhood cancer’ was attended by 14 participants from 8 Francophone countries: Burkina Faso, France, Ivory Coast, Mauritania, Morocco, Niger and Senegal. The objective was to provide skills to assist healthcare workers to decide when to use palliative or curative protocols for patients. This was based on the findings that in Francophone Africa most patients present with late-stage disease. Advanced disease complicates management, leaving limited options for treatment which may render diagnostics and treatment impossible in resource-limited settings.

Discussion points included the clarification of the definition of ‘advanced stage’ and ‘late stage’ since it is not related to standardised international staging systems, but can relate to a combination of factors such as tumour volume, metabolic or nutritional status and the degree of tolerance of toxic treatment.

Possible solutions included:

1. To differentiate between three categories of patients:
  - Category 1: patients presenting with a known poor prognosis or patients with a very poor or no chance of survival (DIPG, metastatic alveolar RMS, osteosarcoma with bone metastases, etc.);
  - Category 2: patients presenting with a disease where outcomes have been improved by cellular therapies, targeted therapies or innovative therapies (metastatic neuroblastoma);
  - Category 3: curable diseases focusing on the WHO GICC index cancers.
2. Upon admission each patient should be classified in one of these categories and the treatment tailored accordingly:
  - Category 1: managed with a palliative strategy
  - Category 2: If possible, refer to a local or international centre where therapies are available. If this is not possible, then category 1 treatment (palliative strategy ± metronomic chemotherapy) should be started;
  - Category 3: adapted management guidelines and regimens or standard therapies should be started.
3. Ethical issues raised by inequality of access to care worldwide and how to communicate with families regarding limited resources were discussed.

The final discussions related to the importance of palliative care in LMICs. The number of patients requiring palliative care in LMICs exceeds those in HICs and therefore teams treating childhood malignancies should as a first step set up a palliative care programme parallel to curative strategies. Once management teams gain experience, the number of patients undergoing curative treatment is expected to increase progressively. This strategy should be modulated according to the local setting and the context of resources available in the setting. At the end of the workshop, all the participants acknowledged the importance of a robust classification system to prevent waste of resources that are already limited.

## Discussion

Global resources for paediatric oncology are unequally distributed [35]. While countries like Egypt and South Africa are spearheading bone marrow transplantation and targeted therapies, Burundi and Niger are initiating services and Mauritius has no paediatric oncologists.

Since the launch of the 2018 WHO GICC, Africa has prioritised the principles of the initiative. Morocco, Ghana and Uganda independently supported greater government involvement at all levels of systems development for childhood cancer care: independent paediatric oncology cancer care plans, childhood cancer registries, securing access to chemotherapy supplies and developing surgery and radiotherapy services.

Education programmes were a focus of many free papers’ presentations, reflecting the current need in Africa. Inaugural workshops (pharmacy, IPSO, radiotherapy and Young SIOP Africa Education Day) and those addressing priority areas (adapted management guidelines, nursing, paediatric palliative care and nutrition) focused on building capacity [36–38]. Africa has shown its ability to adapt, while innovation is growing.

In line with the six priority cancers of the WHO GICC, many presentations focused on retinoblastoma, HL, ALL and nephroblastoma, with more challenging tumours like rhabdomyosarcoma and neuroblastoma receiving limited exposure. Similarly, bone tumours and rare tumours received little attention, while multidisciplinary management of brain tumours is still in its infancy in some countries.

The GICC should receive greater attention moving forwards in North-South African collaborations [39]. Although the GFAOP and CANCaRe groups presented multinational projects, demonstrating the value and feasibility of collaboration, a large number of abstracts focused on single institution studies. HIC collaborators like WCC have led the way in this regard. Hopefully, this approach will be applied increasingly in Africa with the GICC as guiding principles even independent from direct WHO involvement [40].

Civil society (NGOs, parent groups and commercial groups) in the African context will play a large role in financing, managing and advocacy towards reaching the goals of the GICC in Africa in partnership with governments. Africa faces unique challenges related to political instability, internally displaced persons, refugees and diseases like tuberculosis and HIV that are largely managed by NGOs, which remain an important link between centres of expertise and the community [41, 42].

## Conclusion

The 14th SIOP Africa Continental conference delivered in hybrid mode gave an opportunity to reach more people who may not have attended due to concerns about the pandemic. Delegates were enthusiastic about sharing their experiences and innovative approaches in paediatric oncology after two challenging years of COVID-19. This year’s conference featured a record number of pre- and post-conference workshops, reflecting the excitement at being able to participate in person once more. The conference highlighted the need for greater collaborative efforts to consolidate resources for improved childhood cancer outcomes and increased involvement of government to support efforts to achieve the WHO 2030 GICC goals. Whether countries have been officially named as WHO focus countries in the GICC movement or not, the commitment to alignment with these goals was evident in plenary sessions, free papers and discussions. The latest conference of SIOP in Africa rekindled enthusiasm in a depleted but ultimately resilient African paediatric oncology community.

## Abbreviations

AMCC: Alliance Mondiale Contre le Cancer
BL: Burkitt lymphoma
CAR: Chimeric antigen receptor
GFAOP: Francophone African Group of Paediatric Oncology
GICC: Global Initiative on Childhood Cancer
GNI: Gross national income
HL: Hodgkin lymphoma
ICPCN: International Children’s Palliative Care Network
IIPAN: International Initiative for Paediatrics and Nutrition
IPSO: International Paediatric Surgery Organisation
LMIC: Low- and middle-income countries
POU: Paediatric oncology unit(s)
PROS: Paediatric radiation oncology society
RT: Radiotherapy
SIOP: International Society of Paediatric Oncology
UCI: Uganda Cancer Institute
UNCCP: Uganda National Cancer Control Programme Plan
WCC: World Child Cancer
WHO: World Health Organisation

## Conflicts of interest

The authors declare that they have no conflict of interests.

## Funding

Funding for the publication of this report was provided by the Paediatric Oncology Department, Antwerp University Hospital, Antwerp, Belgium.

## Figures and Tables

**Table 1.** SIOP Africa 2022 awards [6].

Award	Recipient
Friend of SIOP Africa	Prof Catherine Patte (France) for her contributions to the continent through her work with the GFAOP.
Lifetime Achievement Award: Medical	Prof Elhamy Rifky (Egypt) for his dedication to improving services in Egypt and support of African and Middle Eastern colleagues.
Lifetime Achievement Award: Nursing	Sr Enyo Bosumprah (Ghana) for mentoring and educating nurses in Ghana, and her many contributions to local and international paediatric oncology organisations.
Best oral presentation	‘Association between high-risk histopathological and clinical features of primary enucleated eyes at Muhimbili National Hospital’. N. Moshi, Muhimbili University of Health and Allied Services, Dar es Salaam, Tanzania.
Best poster presentation	‘Combatting treatment dropout in paediatric cancer’. R. Kabore, Yalgado Ouedraogo University Hospital, Ouagadougou, Burkina-Faso
Most innovative presentation	‘Integrating a palliative approach into the healthcare provided by paediatric oncology units. Insights from a 3-year training programme’. C. Edan, GFAOP - Gustave Roussy, Villejuif, France

**Table 2.** SIOP Africa 2022 various meetings and proceedings [6].

Pre-conference days			Conference days		Post-conference day
**Day 1**	**Day 2**	**Day 3**	**Day 1**	**Day 2**	**Day 1**
AMCC Retinoblastoma workshop Day 1	AMCC retinoblastoma workshop Day 2	Civil Society and Parents Symposium	**OPENING REMARKS**	**PLENARY SESSION** Targeted therapy	Nutrition workshop
Wilms’ tumour workshop Day 1	Wilms’ tumour workshop Day 2	Nurses children’s palliative care workshop	**PLENARY SESSION** WHO GICC	**PARALLEL SESSIONS** Presentation of abstracts	
	Radiation oncology workshop	Paediatric oncology pharmacy workshop	**CONFERENCE OPENING**	**NURSES BUSINESS** *Meeting*	
	Young SIOP educational day	IPSO Surgical Symposium	WCC workshop	SIOP AFRICA **ANNUAL BUSINESS** *Meeting*	
	**GFAOP** *Meeting*	The ARIA-adapted management guidelines workshop	**PARALLEL SESSIONS** Presentation of abstracts	**PARALLEL SESSIONS** Presentation of Abstracts	
			**PLENARY SESSION** Advocacy for access	**PLENARY SESSION** Childhood cancer in special circumstances	
